# A sound-driven cortical phase-locking change in the *Fmr1* KO mouse requires *Fmr1* deletion in a subpopulation of brainstem neurons

**DOI:** 10.1016/j.nbd.2022.105767

**Published:** 2022-05-17

**Authors:** Andrew J. Holley, Aleya Shedd, Anna Boggs, Jonathan Lovelace, Craig Erickson, Christina Gross, Miranda Jankovic, Khaleel Razak, Kimberly Huber, Jay R. Gibson

**Affiliations:** a University of Texas Southwestern Medical Center at Dallas, Department of Neuroscience, Dallas, TX 75390-9111, USA; b Division of Child and Adolescent Psychiatry, Cincinnati Children’s Hospital Medical Center, Cincinnati, OH 45229, USA; c Department of Psychology, University of California, Riverside, CA 92521, USA; d Department of Psychiatry, University of Cincinnati College of Medicine, Cincinnati, OH 45229, USA; e Division of Neurology, Cincinnati Children’s Hospital Medical Center, Cincinnati, OH 45229, USA; f Department of Pediatrics, University of Cincinnati College of Medicine, Cincinnati, OH 45229, USA

**Keywords:** Fragile X syndrome, Brainstem, Inferior colliculus, Electroencephalography, EEG, Auditory, Timing, Phase-locking, Sensory responses, Autism

## Abstract

**Background:**

Sensory impairments commonly occur in patients with autism or intellectual disability. Fragile X syndrome (FXS) is one form of intellectual disability that is often comorbid with autism. In electroencephalographic (EEG) recordings obtained from humans with FXS, the ability of cortical regions to consistently synchronize, or “phase-lock”, to modulated auditory stimuli is reduced compared to that of typically developing individuals. At the same time, less time-locked, “non-phase-locked” power induced by sounds is higher. The same changes occur in the *Fmr1* knockout (KO) mouse – an animal model of FXS. We determined if *Fmr1* deletion in a subset of brainstem auditory neurons plays any role in these EEG changes in the mouse.

**Methods:**

We reinstated FMRP expression in a subpopulation of brainstem auditory neurons in an otherwise *Fmr1* KO control (conditional on; cON *Fmr1*) mouse and used EEG recordings to determine if reinstatement normalized, or “rescued”, the phase-locking phenotype observed in the cON *Fmr1* mouse. In determining rescue, this also meant that *Fmr1* deletion in the same neuron population was necessary for the phenotype to occur.

**Results:**

We find that *Fmr1* reinstatement in a subset of brainstem neurons rescues certain aspects of the phase-locking phenotype but does not rescue the increase in non-phase-locked power. Unexpectedly, not all electrophysiological phenotypes observed in the *Fmr1* KO were observed in the cON *Fmr1* mouse used for the reinstatement experiments, and this was likely due to residual expression of FMRP in these *Fmr1* KO controls.

**Conclusions:**

*Fmr1* deletion in brainstem neurons is necessary for certain aspects of the decreased phase-locking phenotype in the *Fmr1* KO, but not necessary for the increase in non-phase-locked power induced by a sound. The most likely brainstem structure underlying these results is the inferior colliculus. We also demonstrate that low levels of FMRP can rescue some EEG phenotypes but not others. This latter finding provides a foundation for how symptoms in FXS individuals may vary due to FMRP levels and that reinstatement of low FMRP levels may be sufficient to alleviate particular symptoms.

## 1. Introduction

FXS is a neurodevelopmental disorder caused by loss of function mutations in the X-linked *FMR1* gene which encodes Fragile X Mental Retardation Protein (FMRP), an RNA binding protein. Many of the impairments in FXS are reproduced in the FXS mouse model, the *Fmr1* KO mouse (Bakker, 1994). Humans with FXS have increased magnitude of auditory responses as observed in fMRI, MEG and EEG studies (Rotschafer and Razak, 2014; Rais et al., 2018; Ethridge et al., 2016; Castren et al., 2003; Van der Molen et al., 2011). These response magnitude increases correlate with sensory hypersensitivity and communication deficits in FXS patients, indicating that these EEG alterations are associated with clinical outcomes (Ethridge et al., 2016). *Fmr1* KO mice have analogous enhanced auditory responses, as observed by event-related potential (ERP) amplitude and the firing of individual neurons located in the cortex, lateral superior olive, and inferior colliculus (IC) (Nguyen et al., 2020; Rotschafer and Razak, 2013; Garcia-Pino et al., 2017).

Recent studies describe two EEG phenotypes that are observed in both FXS individuals and *Fmr1* KO mice. These involve responses to a “chirp” stimulus. First, sound-driven phase-locking is reduced which reflects an impairment in the ability of cortical activity to be consistently and repeatedly time-locked to sounds (Ethridge et al., 2017; Wang et al., 2017; Lovelace et al., 2016; Lovelace et al., 2018). Second, there is higher non-phase-locked gamma power during the chirp (Ethridge et al., 2017; Wang et al., 2017; Lovelace et al., 2016; Lovelace et al., 2018). Non-phase-locked power includes both phase-locked power and power contributed by response components that are not phase-locked (or nonspecifically induced). The differing degrees of these two EEG alterations among FXS individuals correlate with cognition, sociability, and performance on behavioral tests (Ethridge et al., 2019). Therefore, both changes are potential biomarkers for FXS which might be used to diagnose and assess treatment outcomes. The specific mechanisms and circuits underlying these EEG alterations remain unclear and can be studied in *Fmr1* mouse models.

While the surface-based EEG signal is mostly generated by neocortex, this does not mean that the changes in the EEG of FXS individuals occur only in the cortex. Sound processing begins in the cochlea and proceeds through key subcortical nodes before reaching the cortex. Therefore, circuit alterations in any number of brain regions could underlie the sound-driven phase-locking decrease or the non-phase-locked power increase (McCullagh et al., 2020a). The increased non-phase-locked power is likely caused by *Fmr1* deletion in cortex (Lovelace et al., 2020a; Rais et al., 2021). But *Fmr1* deletion in cortex appears to play a smaller role in the phase-locking phenotype (Lovelace et al., 2020a; Rais et al., 2021). Therefore, deletion in other brain regions likely plays a role in the phase-locking decrease, but these remain unknown. Also unknown is whether the sound-driven phase-locking deficit is mediated by changes in different circuits depending on stimulus parameters such as frequency. Resolving these unknowns would be the first step in understanding the specific pathological mechanisms and developing potential treatment strategies for improving auditory processing in Fragile X syndrome.

Auditory brainstem circuits mediate easily measured sensory evoked behaviors and physiological responses, and therefore represent a promising translational model system for studying neurodevelopmental disorders such as FXS. FMRP is strongly expressed in the brainstem (medulla/pons/midbrain) (Zorio et al., 2017). In the midbrain, *Fmr1* mRNA levels are high early in development (P14) and decline into adolescence (decreased by ~66% at P28) (Yun et al., 2006). Electrophysiological and anatomical changes are observed in auditory nuclei in the pons and medulla of the *Fmr1* KO mouse (Garcia-Pino et al., 2017; Rotschafer and Cramer, 2017; Rotschafer et al., 2015; Brown et al., 2010; Strumbos et al., 2010; El-Hassar et al., 2019; McCullagh et al., 2017) and in analagous structures of the zebrafish *Fmr1* KO (Constantin et al., 2020), but how brainstem changes specifically impact auditory processing at a systems level remains unclear. Changes in IC circuitry may underlie auditory processing deficits since sensory responses and population encoding of stimuli are altered in this region in the *Fmr1* KO (Nguyen et al., 2020; McCullagh et al., 2020b; Kokash et al., 2019). Moreover, both FXS individuals and *Fmr1* KO mice have altered brainstem-mediated acoustic reflexive behaviors, such as pre-pulse inhibition (PPI) of acoustic startle (McCullagh et al., 2020b; Frankland et al., 2004; Hessl et al., 2009; Koch, 1999; Fendt et al., 2001). We hypothesize that brainstem circuits, particularly those involving the IC, are a locus for changes that alter auditory responses in the cortical EEG of *Fmr1* KO mice.

We determined whether *Fmr1* deletion in brainstem auditory neurons was necessary for two EEG phenotypes described above and observed during a chirp stimulus - decreased sound-driven phase-locking and increased non-phase-locked power in the gamma frequency band. We targeted a subpopulation of brainstem neurons – “*Ntsr1*-marked” neurons - which consisted mostly of IC glutamatergic neurons and to a much lesser extent, some putative glutamatergic neurons in the cochlear nuclei (Gale and Murphy, 2014; Faingold, 2002; Saul et al., 2008; Ito et al., 2011). This study provides important insights into the mechanisms underlying altered sensory processing in FXS individuals and into the biological basis of promising clinical biomarkers for FXS.

## 2. Methods

### 2.1. Mice

For conditional expression experiments, male *Ntsr1*-Cre mice (*Ntsr1*^Cre/+^ mice; GN209, GENSAT) (Gong et al., 2007; GENSAT, 2003) were crossed to female conditional ON (cON or cON *Fmr1*) mice (*Fmr1*^loxP-Neo/y^ mice) (Mientjes et al., 2006; Guo et al., 2011). These mice were on the C57bl/6 J background. The cON *Fmr1* mice are considered functionally equivalent to *Fmr1* KO mice and conditionally express *Fmr1* when Cre-recombinase is present. One caveat with cON *Fmr1* mice is that the allele is “leaky” and expresses *Fmr1* at 5% to 15% wild-type (WT) levels ( (Guo et al., 2011) and personal communication from Dr. David Nelson of Baylor College of Medicine, respectively). Images illustrating expression of Cre in the brain of *Ntsr1*-Cre mice are at the GENSAT website (GENSAT, 2003), and they indicate strong somal expression in the IC and fiber expression in the medial geniculate of the thalamus due to projections from the IC (at (GENSAT, 2003), images 58 and 69. Note image 63 is out of order and should be 59). We observe clear Cre-dependent YFP-reporter expression in brainstem at postnatal day 0 (P0) indicating that Cre-expression begins embryonically (Supp. Fig. 1), but the specific time during embryogenesis is not known.

Male progeny, age P50–60 from *Ntsr1*-Cre and cON crosses could be any one of 4 genotypes based on expression of the 2 alleles: 1) None (WT), 2) Cre only, 3) cON only, and 4) both (Cre:cON). The first 2 were combined into a single genotypic group named “WT controls”. This grouping was acceptable because we have never observed effects of Cre-expression using the *Ntsr1*-Cre mouse in this study or in a previous study. The grouping was also necessary in order to acquire enough samples to resolve effects. The cON mice were considered *Fmr1* KO controls. The combination of Cre:cON resulted in reinstatement of *Fmr1* expression in only *Ntsr1*-marked neurons in an otherwise *Fmr1* KO mouse and was called the *Ntsr1*:cON group. Therefore, 3 genotypic groups were compared: WT controls, *Fmr1* KO controls, and *Ntsr1*:cON. We also employed “constitutive” *Fmr1* KO mice (Bakker, 1994) for the same experiments, age P55-60, on a C57bl/6 J background. At this age, accelerated hearing loss in the C57bl/6 J mice is minimal (Spongr et al., 1997; White et al., 2000; Prosen et al., 2003), and because all group comparisons are within-strain (except when noted) and age-matched, hearing loss is unlikely to factor into the phenotypes described here. For a minority of experiments, we used P25-30 FVB mice where *Ntsr1*-Cre and cON lines were backcrossed onto the FVB strain for at least 5 generations. Crossing *Ntsr1*-Cre mice with tdTomato-reporter mice showed identical expression of Cre in the brainstem to that observed in the C57bl/6 J *Ntsr1*-Cre mice (data not shown).

*Fmr1* KO mice (Bakker, 1994), cON *Fmr1* mice, and *Ntsr1*-Cre mice were maintained on a C57bl/6 J background for at least 8 generations when first obtained by the lab, and after that, maintained by interbreeding with WT C57bl/6 J mice obtained from other lines or obtained from Jackson Laboratories. We did this in attempt to best make the genetic background of these lines the same.

Mice were housed in an accredited vivarium on a 12-h light/dark cycle. Food and water were provided ad libitum. Genotypes were determined using PCR analysis of genomic DNA isolated from toe or tail clippings. All procedures were approved by the University of Texas Southwestern Medical Center Institutional Animal Care and Use Committee. Experiments were conducted in accordance with the NIH *Guide for the Care and Use of Laboratory Animals*.

### 2.2. Auditory stimulus presentation

**Fig. 1.**
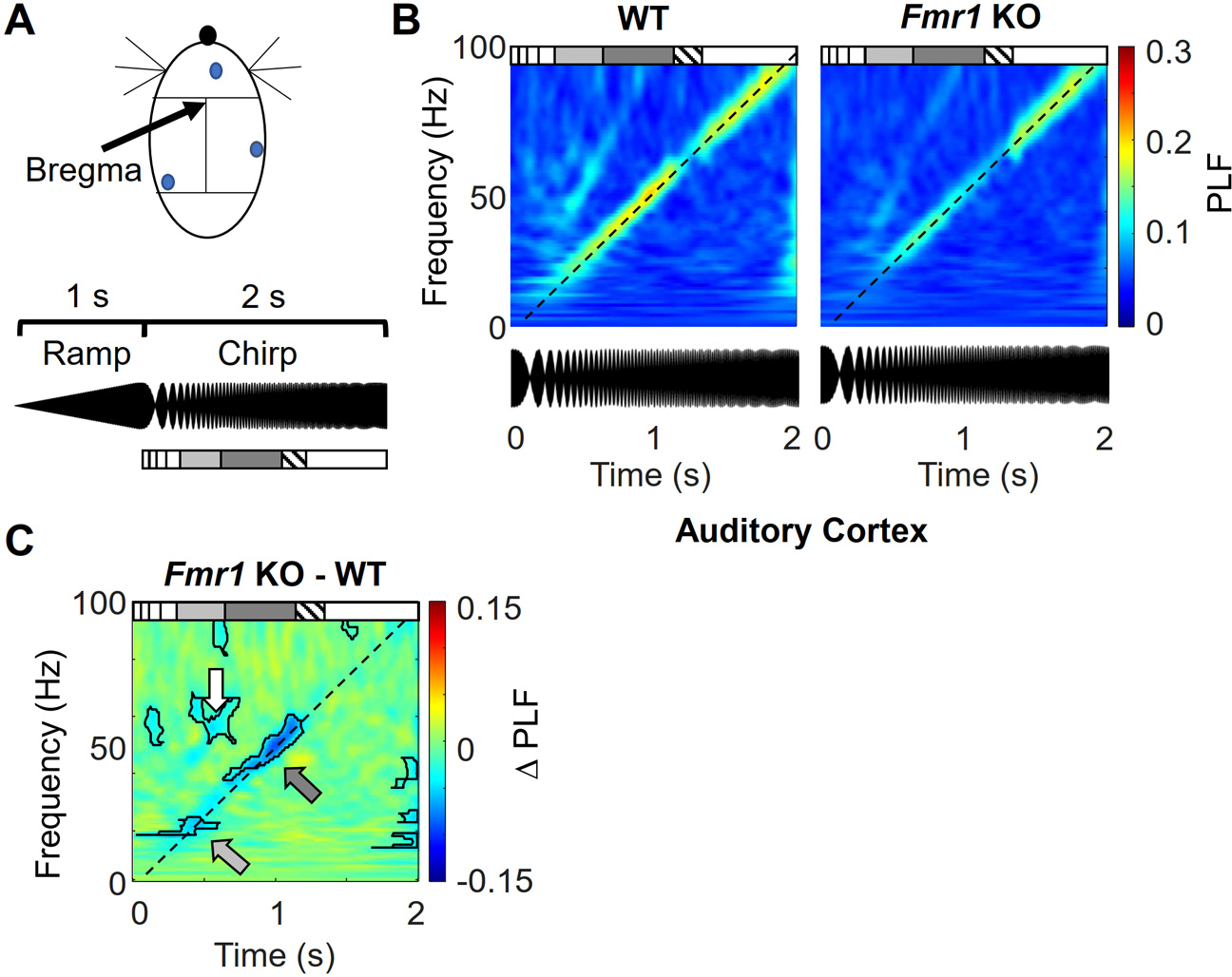
Decreased beta and low gamma frequency phase-locking factor (PLF) in the *Fmr1* KO mouse in auditory cortex. A) Screw electrode placement (*above*) and sound stimulus waveform (*below*) are illustrated. The chirp carrier sound was a 14 kHz tone. The box diagram below the sound waveform marks the time domains of the standard frequency bands corresponding to the current chirp frequency. The light and dark gray boxes mark the time periods for quantifying activity during the beta and low gamma bands, respectively. The hatched box marks the “notch filter” region where signal was removed. B) Average PLF color plots occurring during the chirp. The dashed diagonal line marks the current chirp modulation frequency driving brain activity (see Methods). Below the PLF plots are the chirps aligned to the time axis of the color plots. C) The difference in PLF obtained by subtracting the WT PLF plot from the *Fmr1* KO PLF plot in B. Dark line contours in difference plots border regions of statistically significant differences (*p* < 0.05, this applies to all color difference plots in succeeding figures). The light and dark gray arrows mark regions in the beta and low gamma bands, respectively, that display a statistically significant decrease in PLF. The white arrow highlights the difference in one portion of the second harmonic of the chirp. *n* = 17,20 mice; WT,KO.

Auditory stimuli were generated using Real-Time Processor Visual Design Studio (RPvdsEx) software and delivered using a RZ6 Multi I/O Processor (Tucker Davis Technologies, Alachua, FL). For the presentation of auditory stimuli, a free-field speaker (MF-1 speakers, TDT, Alachua, FL) was mounted ~12 in. directly above a cage with fresh bedding. This was housed inside a chamber (Expanded PVC S.A.C. w/window, Med Associates, Inc.) surrounded by a custom-made Faraday cage. Unless stated otherwise, sounds were created by the modulation of a 14 kHz carrier tone (70 ± 3 dB SPL in the 5–35 kHz band). Both sound level measurements and recordings to confirm intended generation of waveforms were performed by the same system (CM16/CMPA microphones, 416H 200 amplifier, Avisoft-Bioacoustics) by placing the microphone on the floor of the recording chamber. All sounds were created and presented at a sampling rate of 97,948 samples/s. Chirp protocols are based on those used when sound-driven phase-locking deficits were first observed in the *Fmr1* KO mouse (Lovelace et al., 2018) and in humans with FXS (Ethridge et al., 2017). Briefly, a “chirp” is a stimulus in which the carrier sound amplitude is modulated by a sinusoid whose frequency increases from 1 to 100 Hz (Fig. 1A, *above*). This is preceded by a linear volume ramp to avoid onset responses contaminating phase locking to the amplitude modulation of the chirp (see Fig. 1A). The chirp was repeated for 300 trials. Including the pre-stimulus 1 s and post-stimulus 0.5 s baseline periods for each recorded trial, each trial was 4.5 s long, and the inter-trial interval was 3 s. Previous studies revealed similar phenotypes when the modulation was in an upward (1 to 100 Hz) or downward (100 to 1 Hz) direction (Lovelace et al., 2018). Therefore, just the upward modulation was studied here.

In the conditional expression experiments, we recorded event related potentials (ERP). Stimuli consisted of a sound pulse train of four broadband white noise “clicks” (100 ms duration, separated by 4 s with a 4 s interval between each train, 70 ± 3 dB SPL). This stimulus train was repeated 100 times. Using PLF and non-PLF measurements of the responses (described in methods below), we observed no differences among groups and these were not investigated further (data not shown; *n* = 29,15,12).

### 2.3. Electroencephalogram (EEG) recordings

For EEG recordings, we performed electrocorticography (ECoG) as described previously (Lovelace et al., 2016; Lovelace et al., 2018). Recordings were made from screw electrodes which were implanted into the skull, touching the dural surface over frontal and auditory regions of the cortex (see location details below). Mice were habituated for 15–20 min after being brought into the EEG recording room. The mice were then placed in an anechoic foam-lined soundproof chamber and tethers were connected to the implanted posts through a 3-channel tether that was passed through a commutator located directly above the recording cage. Mice were habituated to being tethered to the commutator for an additional 15–20 min. The tethers were connected to a 16-channel S-Box channel splitter which fed data through a PZ3 low impedance amplifier (Tucker-Davis Technologies, TDT, Alachua, FL). The EEG data is recorded through a RZ2 Bio Amp Processor (TDT, Alachua, FL). After habituation, at least 5 min of resting EEG were recorded in the absence of auditory stimuli. Next, the mice were presented with 300 presentations of a chirp-modulated tone (1–100 Hz, 2 s). After recordings were completed, mice were returned to the colony.

The lead to the occipital cortex served as a reference electrode for the frontal and auditory cortex screws. Acquisition hardware was set to high-pass (>0.5 Hz) and low-pass (<150 Hz) filters. Data were sampled at a rate of 610.35 Hz via OpenProject software (TDT, Alachua, FL).

Movement is known to elevate cortical gain during EEG recordings (Niell and Stryker, 2010). To account for movement, we placed a piezoelectric transducer below recording cages to track movement during recordings. Prior to collecting experimental data, the activity from the piezoelectric transducer was evaluated in conjunction with video (RV2 Video Capture System, TDT, Alachua, FL) to confirm threshold criteria for movement. The EEG segments in which movement was occurring were rejected and excluded from analysis for resting EEG recordings and non-phase locked power during the chirp presentations.

### 2.4. Surgery for EEG recordings

Mice were anesthetized using 1–2% isoflurane and this was maintained through a nose-mask attached to a stereotaxic frame (Narishigi Group, Japan). Artificial tears were used to keep the eyes moist during anesthesia and doses of buprenorphine sustained release (SR) and carprofen were administered subcutaneously. Toe/tail pinch reflexes were monitored throughout. Once anesthetized, a midline incision was made in the scalp to expose the skull. A precision micro-drill (Fine Science Tools, Foster City, CA) was used to create three 0.9 mm holes over the right frontal cortex (+3.0 mm, +1.6 mm | Coordinates relative to bregma: anterior/posterior, medial/lateral), right auditory cortex (−1.6 mm, +4.8 mm), and the left occipital (−4.2 mm, −5.1 mm) (Fig. 1B). A 3-channel post (MS333-2-A-SPC, P1 Technologies, Roanoke VA) was connected to screws (8L003905201F, P1 Technologies) which were placed into the drilled holes until they contacted the dura mater. Dental cement (Panavia SA cement plus, Kuraray America, Houston, Texas) was applied to cover the screws, base of the 3-channel post, and skull. Hardening of the cement was expedited using a LED curing light. Mice were then placed on a circulating water heating pad and given 0.5 mL saline to aid in recovery. After recovery from surgery, the mice were individually housed and were returned to the vivarium and monitored daily until the day of recordings. Additional doses of carprofen were given 24 h and 48 h post-surgery. Mice were given between three to five days of post-operative recovery time and recordings were conducted between P50-P60 (C57Bl/6 J) or between P25-P30 (FVB, “4 week old”).

### 2.5. Data analysis

EEG data were analyzed in custom LABVIEW software (National Instruments Corp, Austin, TX). Data were pre-processed using a 0.5–150 Hz bandpass filter. A 60 Hz notch filter was applied. All recordings were cleaned for artifacts using a semi-automatic procedure in LABVIEW. A similar semi-automatic procedure was used to define movement based on signals from piezoelectric transducers.

Resting EEG data was collected continuously for 5 min with no experimental stimulus presentation. Recordings were divided into 1 s length segments and underwent a Fast Fourier Transform (FFT) to obtain a power spectrum (μV/Hz^2^) from 1 to 100 Hz. Power was then summed into standard frequency bands: Delta (1–4 Hz), Theta (4–8 Hz), Alpha (8–13 Hz), Beta (13–30 Hz), Low Gamma (30–55 Hz), and High Gamma (65–100 Hz). We only used EEG segments during which a mouse was immobile.

### 2.6. Phase-locking factor (PLF)

For each mouse, traces from individual trials involving sound stimuli (e.g. chirp) underwent a Morelet wavelet transformation, *F*_*k*_*(f,t),* which refers to the complex wavelet coefficient at a given frequency and time for the *k*th trial. The PLF as a function of time and frequency was derived from calculating intertrial phase coherence (ITPC). The Morelet wavelet transforms from all trials were used to calculate the ITPC as follows:

$$\mathrm{ITPC}(f,t)=\frac{1}{n}\sum_{k=1}^{n}\frac{F_{k}(f,t)}{\left|F_{k}(f,t)\right|}$$

where *f* is the frequency, *t* is the time point, and n is total trial number. We used a peak frequency to width ratio of 15. Phase-locking data from a mouse was accepted for analysis only if there was a response to brief sound pulses. A response was considered absent if the response, as measured by the background subtracted non-phase-locked power, was less than 2 standard deviations above baseline at the peak time and frequency of the response. Inspection of the data indicated weak or no phase-locking when response to a brief pulse was absent.

As shown previously, we found that at any given timepoint in the average PLF plots, the peak frequency corresponded to the modulation frequency of the chirp occurring slightly prior to that time. In our experiments, this offset was approximately 50 ms. This information was used to define a region of interest (ROI) for examining our results (diagonal lines in Fig. 1B,C).

### 2.7. Single Trial non-phase-locked (non-PL) power

Single trial non-PL power as a function of time and frequency was calculated by obtaining a time-series power spectrum. This was done by extracting absolute values from complex values obtained from the Morelet wavelet transformation (squareroot[real^2^ + imaginary^2^]) for each cell in each trial matrix. Absolute value matrices were then averaged for all trials for a given mouse, and group grand average matrices were then compiled for each group. For analysis, data are binned into the standard frequency bands and averaged across the relevant time window. For chirp, the time window was during the 2-s chirp.

Single trial non-PL power was used to measure baseline power and to measure response magnitude to sound stimuli. For the latter, the response was measured during the stimulus and calculated relative to the non-PL power existing in the 1 s baseline period immediately preceding the sound stimulus.

### 2.8. Slice electrophysiology

We measured the duration of spontaneously occurring activity bursts in primary somatosensory cortex in acutely prepared slices obtained from cON and WT mice (age P20-23). Methods for this are described in our previous publication (Gibson et al., 2008).

### 2.9. Immunoassay for quantification of FMRP

#### 2.9.1. Sample preparation

Cortex and brainstem samples were obtained from mice (P55-60) on the C57bl/6 J background and belonging to one of three genotypic groups - (cON, *Fmr1* KO, and WT). Brains were dissected and immediately frozen on dry ice at UT Southwestern. All samples were shipped on dry ice to Cincinnati Children’s Hospital Medical Center. Tissue samples were lysed with elution buffer (M-PER with salt, Antipain, Chymostatin, Protease Inhibitor) and manually homogenized. The lysates were centrifuged, and the supernatant was collected for immediate use in the assay. Total protein concentration was determined via the Pierce Rapid Gold BCA Protein Assay (ThermoFischer Scientific, A53226). Pilot studies showed that 12 μg of total protein of a known cortical wildtype (WT) sample measured the upper limit of detection of the Luminex assay. To ensure that all samples would fall within the detection limits, we loaded 6 μg of cortical and 6 μg brainstem into each assay well at a volume of 50 μL.

Trunk blood was collected into individual 1.5 mL low protein-binding microfuge tubes containing 10 uL of K2EDTA and snap frozen. Blood samples were shipped on dry ice to Cincinnati Children’s Hospital Medical Center. From the thawed collection tube, 50uL of blood was spotted onto ID Bloodstain Cards (Whatman Bloodstain Cards, WB100014) and allowed to dry for 4 h. Three dried blood spots (DBS) were collected from each card using a 6 mm hole punch and transferred into CoStar Spin-X Centrifuge Filter Tubes (7200388) with elution buffer. Proteins were extracted from the DBS overnight at room temperature with orbital shaking. The eluates were collected after a 6-min centrifugation at 12000 ×*g* and immediately used in the assay. 50 μL of the eluate was used per well in the assay.

#### 2.9.2. Immunoassay procedure

A standard range of fluorescence was generated using known WT samples for each sample type (cortex, brainstem, DBS). A 1:2 dilution series was performed using the known WT sample for a total of 9 standard points ranging from 12 to 0.05 μg of total protein. The immunoassay procedure was performed as previously described (Budimirovic et al., 2020; LaFauci et al., 2013). Briefly, a total volume of 50 uL of sample was aliquoted into assay wells of a 96-well low protein binding plates (Greiner Bio-One, 655,096). Luminex Magspheres were coupled to a concentrated capture antibody, mAb 5C2 (BioLegend, 834,701) according to manufacturer’s instructions. Magsphere beads diluted to 80 beads/uL were aliquoted into assay wells at a volume of 50 uL to bring the total well volume to 100uL. After a 6-h incubation on a plate shaker, the beads were washed manually in assay buffer (PBS, 1% BSA, 0.05% Tween-20). The plates were incubated overnight at 4 °C in secondary detecting antibody (Abcam, ab17722). After manual washing, the plates were incubated at room temperature for 2 h in signal detecting antibody (Jackson ImmunoResearch, 711-116-152). Plates were vigorously washed and resuspended in 100 μL of sheath fluid (Luminex, 40–50,021). The magspheres were analyzed (in triplicate) on the Luminex 200 system to determine median fluorescence intensity.

#### 2.9.3. FMRP protein expression quantification

Individual FMRP concentration was determined by generating a fluorescent dilution curve from the known WT sample as a function of median fluorescence intensity via BioPlex Manager Software. Unknown samples were plotted against this curve and reported as a measure of FMRP. Final FMRP value was reported as a percent expression of all WT samples. An Exact Wilcoxon test was first done for the overall statistics on the three groups (cON, *Fmr1* KO, and WT), and this was followed by the Kruskal-Wallis test with FDR (false discovery rate) correction for the pairwise comparisons.

### 2.10. Audiogenic seizures

Methods and analysis were copied from our previous study (Gonzalez et al., 2019).

### 2.11. Statistics

To perform statistical comparisons of PLF and single trial non-PL power data, we performed a nonparametric cluster analysis in MATLAB R2020a (MathWorks, Natick, MA). The PLF and non-PL power data have 2 dependent variables – frequency and time – and consequently, we obtain a 2 dimensional matrix of values from each mouse. The cluster analysis was used to determine contiguous regions in the matrix that were significantly different from a distribution of 1000 randomized Monte Carlo permutations based on previously published methods (Maris and Oostenveld, 2007). In this analysis, if cluster sizes of the real genotype assignments (both positive and negative direction, resulting in a two-tailed alpha of *p* = 0.025) were larger than 97.25% of the random group assignments, those clusters were considered significantly different between genotypes. This method avoids statistical assumptions about the data and avoids the multiple comparison problem. We have used this analysis for the exact same type of EEG data obtained from mice in previous publications (Lovelace et al., 2020a; Rais et al., 2021).

All other statistics were calculated using GraphPad Prism 9 (GraphPad Software, San Diego, CA). For experiments using *Ntsr1*-Cre mice, the following statistics were used. For resting power analysis, the continuous data were binned into standard, discrete frequency bands (in Hz): delta = 1–4, theta = 4–8, alpha = 8–13, beta = 13–30, low gamma = 30–55, and high gamma = 65–100 (Lovelace et al., 2018). A 2-way repeated measures ANOVA was used for resting power frequency band comparisons. If assumptions of sphericity were violated for repeated measures ANOVA, the Greenhouse-Geiser correction was used. For experiments comparing WT and *Fmr1* KO mice, a *t*-test was employed. A *p*-value <0.05 is considered significant for *t*-tests and ANOVAs.

## 3. Results

### 3.1. Sound-driven phase-locking is decreased in beta and low gamma bands in the auditory cortex of the Fmr1 KO mouse

We first examined phase-locking of the EEG to a chirp modulated tone measured at an electrode located over auditory cortical regions (Fig. 1A). We determined if we could reproduce the sound-induced phase-locking decrease observed in the EEG of *Fmr1* KO mice that was reported previously (Lovelace et al., 2018; Jonak et al., 2020). We calculated the phase-locking factor (PLF) as a function of time and frequency during the chirp for both WT and *Fmr1* KO mice and plotted the grand average for each genotype (Fig. 1B). The PLF is a measure of the trial-by-trial consistency of response timing. The strongest PLF during the chirp was, as expected from previous studies, at the frequency of the current chirp modulation indicating that this component of the EEG response was reliably linked to sound features in the 1–100 Hz range (Lovelace et al., 2018). This line of peak PLF is a diagonal across the PLF plot, defining a diagonal ROI (dashed lines in Fig. 1B; see *Analysis* in Methods).

A difference color plot based on the individual group plots illustrates a decrease in PLF in *Fmr1* KO mice at 2 clusters along the diagonal ROI (Fig. 1C). (Lovelace et al., 2020a). One region with decreased PLF in the *Fmr1* KO mouse corresponded to when sound modulation was occurring in the beta band (13–30 Hz) and the other when modulation was occurring in the low gamma band (30–55 Hz) (see arrows in Fig. 1C; average PLFs in the statistically significant beta region for each mouse are plotted in Supp. Fig. 2A). We also observed a decrease in the difference plot corresponding to the second harmonic frequencies corresponding to the boundary of beta and low gamma bands (Fig. 1C, white arrow). Based on previous publications (Fig. 5A,D in (Lovelace et al., 2020b)) and our data here, the other clusters most likely represent either third harmonics of beta- and gamma-related signals (above the ROI) or type I error (below the ROI). Our observation of decreased PLF at specific frequency bands in the *Fmr1* KO confirm previous studies (Lovelace et al., 2018; Jonak et al., 2020).

### 3.2. Rationale for overall experimental design: Determine if Fmr1 deletion in the brainstem is necessary for the decreased sound-driven PLF in the Fmr1 KO mouse

We used the *Ntsr1*-Cre mouse to control *Fmr1* expression in “*Ntsr1*-marked” neurons in the brainstem. A large number of *Ntsr1*-marked brainstem neurons are part of the early stages of auditory processing. This mainly consists of 2 populations – a majority of the glutamatergic neurons in the inferior colliculus (IC) (likely >90%) (Gonzalez et al., 2019), and to a much lesser extent, a small number of putative glutamatergic neurons in the cochlear nuclei (Gale and Murphy, 2014; Faingold, 2002; Saul et al., 2008; Ito et al., 2011; GENSAT, 2003) (Table 1). While these are not the only *Ntsr1*-marked neurons that express Cre, they are the most relevant to our study because they would be expected to directly impact auditory responses in neocortex (more details in *Mice* of Methods and in Discussion). Based on these arguments, this mouse line, together with our use of auditory stimuli, is effective at determining the role of *Fmr1* expression in the brainstem – and in particular, the IC.

To determine if *Fmr1* deletion in *Ntsr1*-marked brainstem neurons is *necessary* for EEG phenotypes, we determined if reinstatement of *Fmr1* in these same neurons could normalize, or “rescue”, the phenotypes. *Ntsr1*-Cre mice were crossed to cON *Fmr1* mice and progeny were divided into 3 genotypic groups: WT controls, cON, and *Ntsr1*:cON. The cON group is a “*Fmr1* KO control”, and the *Ntsr1*:cON mice have reinstatement of *Fmr1* expression in only *Ntsr1*-marked neurons (see *Mice* in Methods for details). The use of the 2 control groups is not always employed for conditional expression experiments and strengthens data reliability and interpretations. In a recent study, we show that the conditional expression of *Fmr1* occurs in the majority of glutamatergic neurons in the IC as predicted (Gonzalez et al., 2019), and for this study, we have observed that FMRP immunostaining in the IC of WT and *Nstr1*:cON mice show comparable cell numbers and intensity levels (data not shown).

If *Fmr1* deletion in *Ntsr1*-marked neurons is necessary for the sound-driven phase-locking impairment in the *Fmr1* KO, we would predict that *Ntsr1*:cON mice would lose the phenotype that is observed in cON mice. The same approach and interpretation has already been successfully used with these same mouse lines to determine the necessity of *Fmr1* deletion in *Ntsr1*-marked neurons for the audiogenic seizure phenotype that exists in *Fmr1* KO mice (Gonzalez et al., 2019).

### 3.3. Fmr1 deletion in Ntsr1-marked neurons is necessary for the sound-driven phase-locking decrease in the beta frequency band observed in the Fmr1 KO mouse

**Fig. 2.**
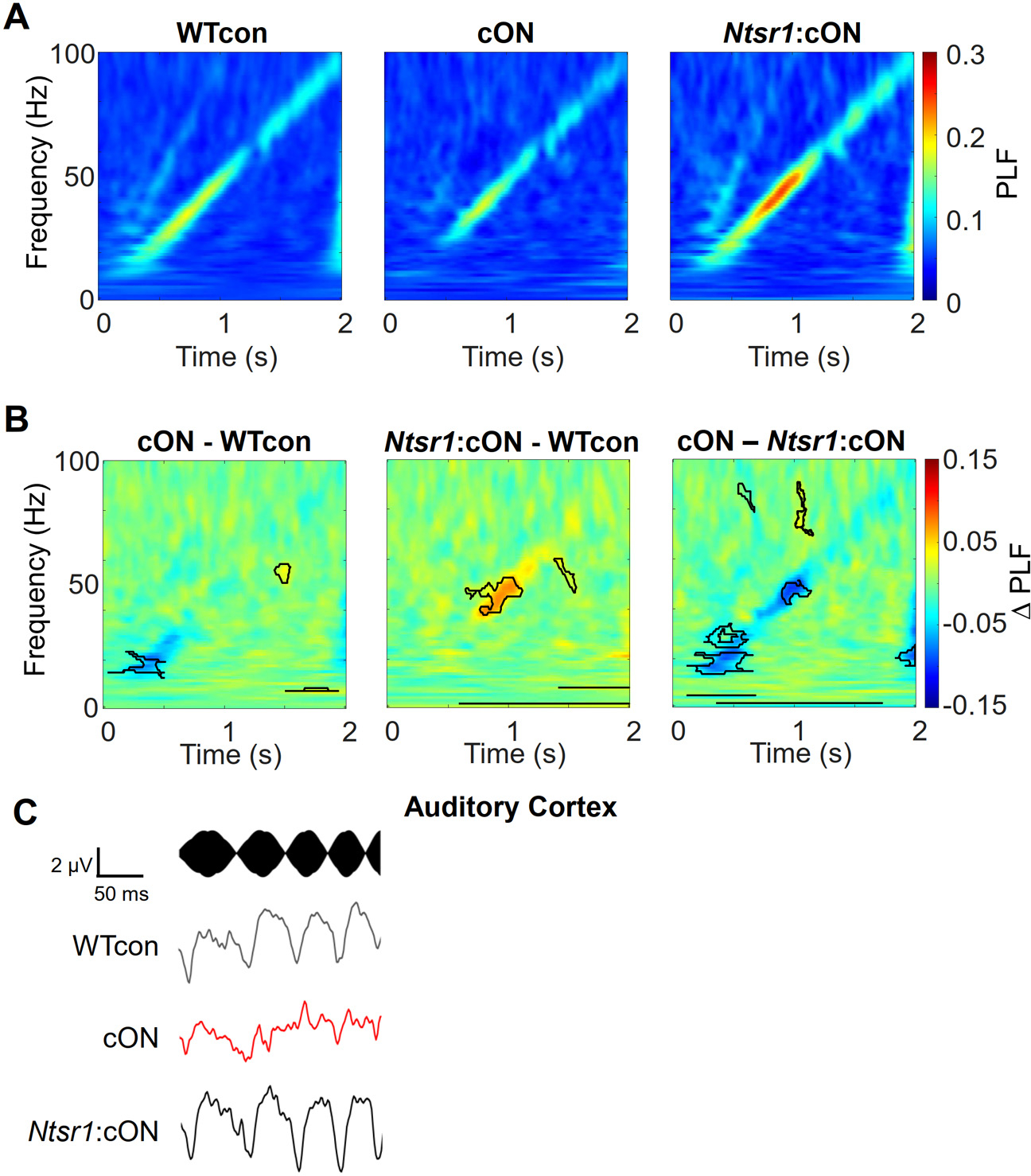
*Re*-instatement of *Fmr1* in *Ntsr1*-marked neurons rescues, or “normalizes”, the decreased PLF in the beta band observed in auditory cortex. A) Average PLF plots of brain activity occurring during the chirp for the 3 genotypic groups. The cON mice are considered *Fmr1* KO controls and *Ntsr1*:cON are mice with *Fmr1* expression only in *Ntsr1*-marked neurons. B) Average PLF difference plots indicate that a decrease in the PLF in the beta band is the most salient phenotype in the cON mice when compared with WT controls (*left*). This decrease disappears with re-instatement of *Fmr1* in *Ntsr1*-Marked neurons (middle). A decrease in the PLF in the beta band is also observed in cON mice when compared with *Ntsr1*-cON mice (*right*). C) The chirp sound (*top*) and average traces of brain activity (*below*) over all mice in the three genotype groups. Only the beta frequency band portion of the chirp and traces is shown. The traces show a decrease in average oscillatory activity in the cON mice which is consistent with the decreased PLF observed at the beta frequency time interval. *n* = 29,15,12 mice; WTcon, cON, *Ntsr1*:cON.

We obtained PLF plots from three genotypic groups for experiments using conditional expression to determine if there is a necessary role for *Fmr1* expression in *Ntsr1*-marked neurons (Fig. 2A). Importantly, we reproduced the decreased sound-driven phase-locking phenotype occurring in the beta band in the cON mice – the *Fmr1* KO controls (Fig. 2B, *left*). This is observed in the difference plot in an almost identical portion of the beta band as the decrease in the constitutive *Fmr1* KO (see Fig. 1C).

Second, selective reinstatement of *Fmr1* in *Ntsr1*-marked neurons eliminated the reduction in beta band PLF. This was observed by the decreased PLF in cON mice compared to *Ntsr1*:cON mice (Fig. 2B, *right*) and the lack of a difference in the beta band between *Ntsr1*:cON mice and WT controls (Fig. 2B, *middle*; average PLFs in the beta region for each mouse are plotted in Supp. Fig. 2B,C). Average traces of chirp responses collected during beta modulation of the sound illustrate the loss of intertrial consistency of phase locking by the decreased modulation amplitude (Fig. 2C). These data indicate that *Fmr1* deletion in *Ntsr1*-marked neurons was necessary for the decreased PLF to chirps during sound modulation in the beta frequency band.

We did not recapitulate the decreased low gamma PLF in cON *Fmr1* mice that we observed in *Fmr1* KO mice (Fig. 2B, *left*). We speculate this may be attributed to the known residual *Fmr1* expression in the cON mouse model (see Methods and Discussion) or to experimental variability. There is also an increase in low gamma power in *Ntsr1*:cON mice compared to WT (Fig. 2B, *middle*), but we don’t pursue this because it does not address a KO phenotype.

### 3.4. No PLF alterations were observed in the auditory cortex of a Fmr1 KO mouse model with a high propensity for audiogenic seizures

We performed identical chirp experiments with identical conditional *Fmr1* expression using younger 4 week old cON mice on a FVB background strain. Arguably, the most robust and reproducible phenotype in *Fmr1* KO mice is the audiogenic seizure (Gonzalez et al., 2019; Musumeci et al., 2000), and deletion of *Fmr1* in *Ntsr1*-marked neurons is necessary for inducing the audiogenic seizure phenotype (Gonzalez et al., 2019). Audiogenic seizures in FVB mice occur at 4 weeks of age. We confirmed that our young cON mice on the FVB background – a *Fmr1* KO model - have audiogenic seizures (8/18 vs. 3/23 mice seized; cON, WT control; *p* < 0.05, Chi-Sqr test). We wanted to know if the above alterations in PLF attributed to loss of *Fmr1* expression in *Ntsr1*-marked neurons were more salient in a *Fmr1* KO mouse model at a time of high propensity for audiogenic seizures, and thereby be possibly related. The use of the FVB strain enabled surgical implants and recordings at younger ages. While strain effects may confound the interpretation of our data, it was still useful to determine how generalized the reduced PLF was across strains and how they relate to the audiogenic seizure.

The cON mice had no detectable changes in PLF compared to WT mice when measured in the auditory cortex (*n* = 17,10,11 mice; WT control,cON,*Ntsr1*:cON; data not shown). Therefore, there was no phenotype in the *Fmr1* KO control to even test the role of *Ntsr1*-marked neurons. In frontal cortex, there was a small decrease in high gamma in the cON mouse, but this was not as compelling as that observed in the *Fmr1* KO and was not rescued in *Ntsr1*:cON mice (Supp. Fig. 3). Because the audiogenic seizure phenotype does exist in FVB cON mice, these negative PLF results suggest that mechanisms underlying the audiogenic seizure are not directly related to changes in the PLF that we observe in this study.

### 3.5. The sound-driven phase-locking phenotype observed in the frontal cortex in the Fmr1 KO is not reproduced in cON mice

**Fig. 3.**
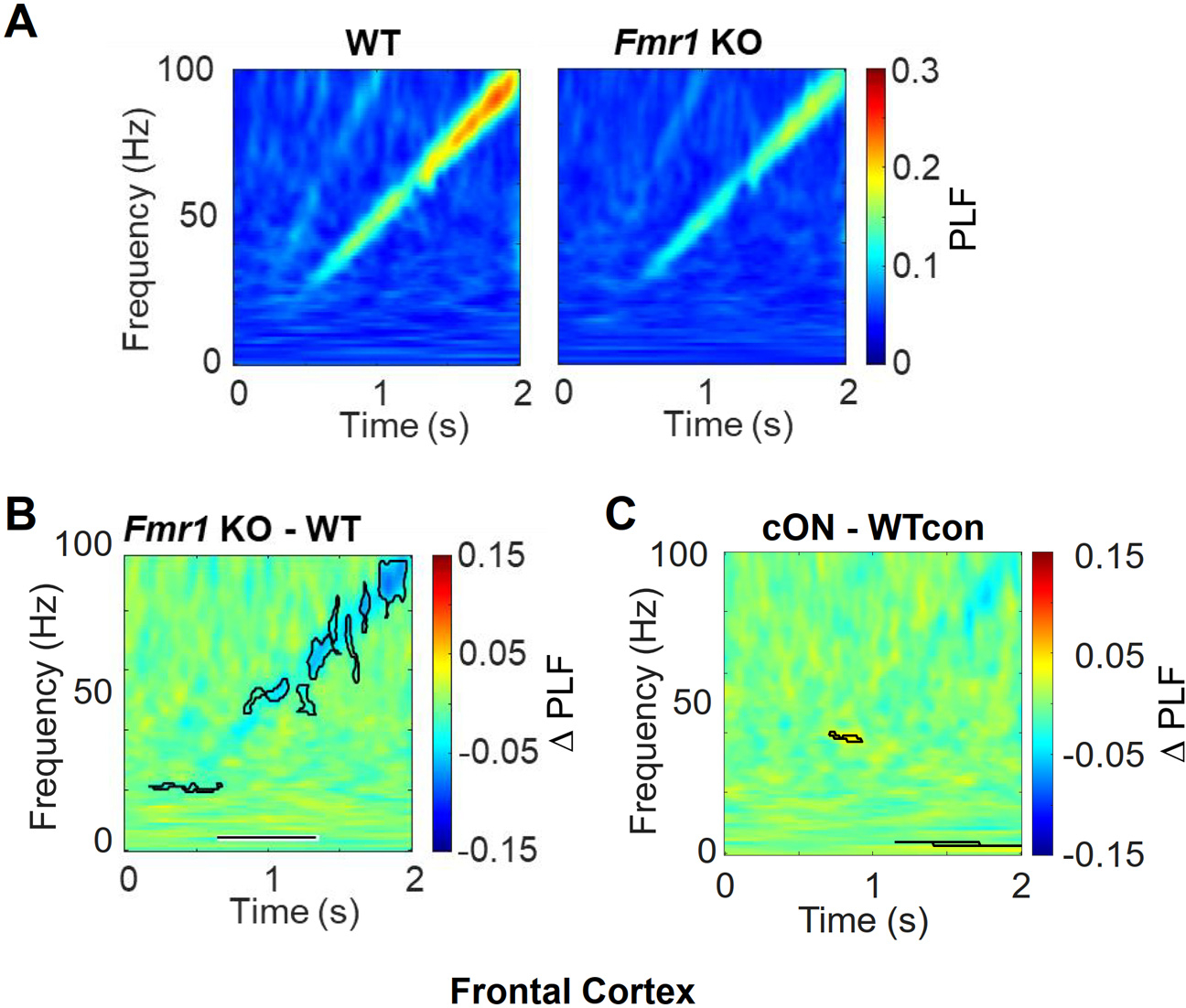
In frontal cortex, the decreased PLF phenotype observed in *Fmr1* KO is not observed in cON mice. A) Average PLF plots of brain activity during the chirp for WT and *Fmr1* KO mice. B) The difference plot of data in A. A decreased PLF is mainly observed at high gamma frequencies in the *Fmr1* KO. C) Average difference plot for cON and WT mice. No notable phenotype was observed in the cON mice. All n’s provided in Figs. 1 and 2.

In the *Fmr1* KO, there was a clear decrease in PLF at gamma band frequencies in the frontal cortex when using the chirp (Fig. 3A,B). This reproduced results from an earlier publication (Lovelace et al., 2018). There was a small, less compelling PLF decrease in the beta frequency band. The clear gamma frequency decrease in the frontal cortex of the *Fmr1* KO using the chirp could not be reproduced in the cON mice (Fig. 3C). Therefore, the cON mouse was not a usable model system to determine the relevant locus for *Fmr1* deletion.

### 3.6. Fmr1 deletion in Ntsr1-marked neurons is not necessary for the increase in non-phase-locked power in the gamma band observed during chirp trials in the Fmr1 KO mouse

In the same mice and recordings described above, we also measured the time series power spectrum of the chirp stimulus trials. We refer to this as the single trial non-PL power, and it includes both “phase-locked” and “non-phase-locked” components relative to the chirp. Previous studies observed increased non-PL power in gamma frequency bands in *Fmr1* KO mice (Lovelace et al., 2018; Jonak et al., 2020; Lovelace et al., 2020b) and have shown that it is due to deletion in the cortex (Lovelace et al., 2020a; Rais et al., 2021). Therefore, we hypothesized that reinstatement of *Fmr1* in the *Ntsr1*-marked neurons would not rescue this phenotype.

**Fig. 4.**
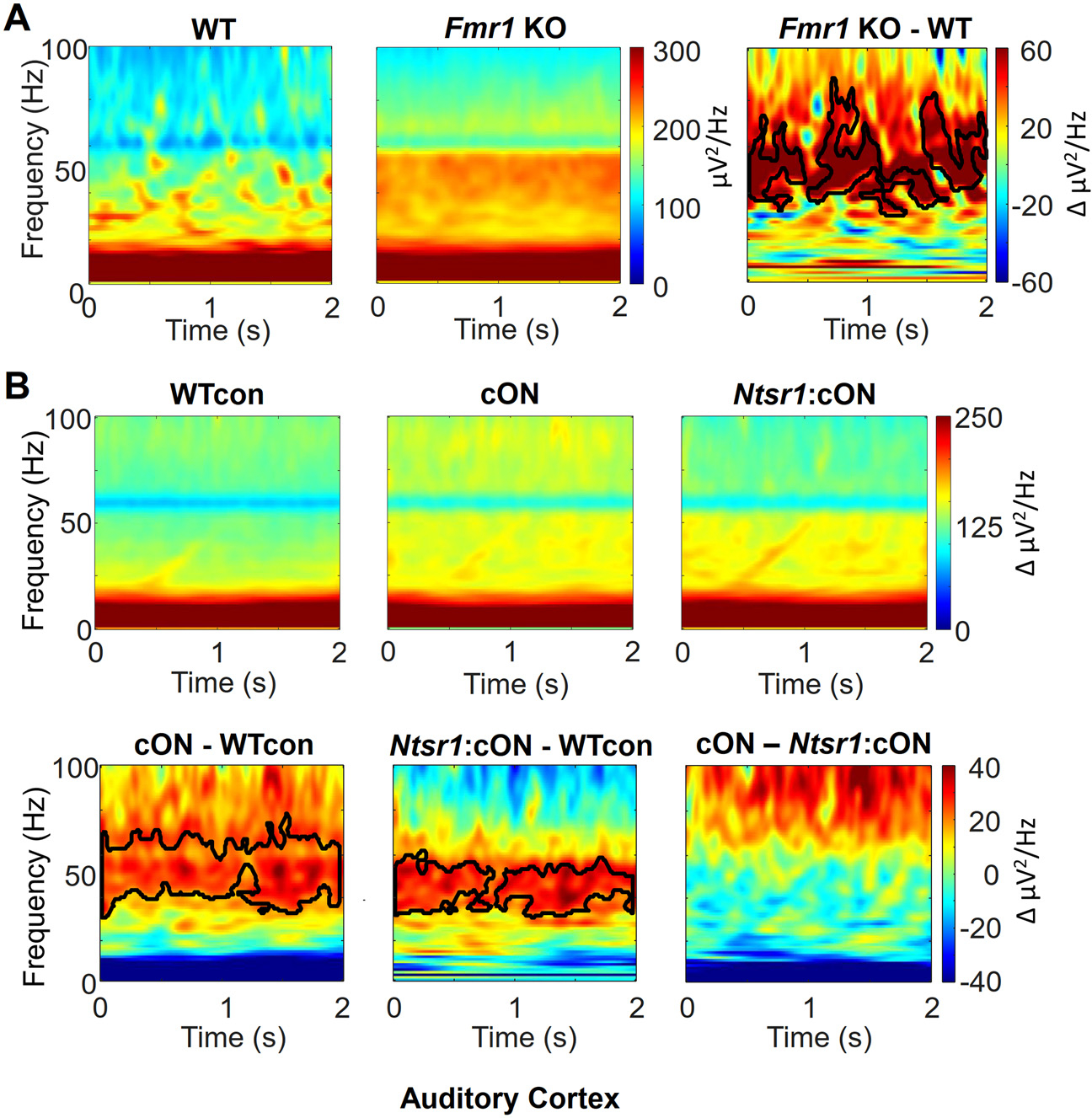
An increase in non-phase-locked power observed in auditory cortex of the *Fmr1* KO was not rescued by *Fmr1* reinstatement in *Ntsr1*-marked neurons. Single trial non-phase-locked power (non-PL) was measured during the chirp. A) Average non-PL power plots (*left*, *middle*) and difference plot (*right*) for WT and *Fmr1* KO mice. An increased non-PL power is observed at gamma frequencies in the *Fmr1* KO. B) Average non PL power plots from auditory cortex (*top*) obtained from conditional expression experiments and non-PL difference plots (*bottom*). The cON *Fmr1* mice had a phenotype of increased power in the low gamma frequency band (*bottom left*) which was not rescued by re-instatement of *Fmr1* in *Ntsr1*-marked neurons (*bottom middle, right*). Dark line contours in difference plots border regions of statistically significant difference. n’s provided in Figs. 1 and 2.

We observed this same gamma band increase in non-PL power in the *Fmr1* KO at both auditory (Fig. 4A) and frontal cortex (Supp. Fig. 4A). This is clearly observed in the non-PL power difference plots in the low gamma frequency range (30–55 Hz, Fig. 4A). This increase in non-PL power was not due to signal induced by the sound since: 1) there was no difference in the small amount of gamma power induced by the ramp/chirp stimulus between *Fmr1* KO and WT mice (4% above baseline, Supp. Figs. 5 and 2) the same increase in non-PL power was observed in the baseline period preceding the ramp/chirp stimuli (data not shown). Therefore, this increase in power was more related to the “background” rather than activity induced by sound stimuli.

We observed the same increase in non-PL power in cON mice in both auditory and frontal cortices (Fig. 4B and Supp. Fig. 4B, respectively). Again, this was mainly in the low gamma range and again was not due to power induced by the sound for the same reasons provided for auditory cortex above (Supp. Fig. 6). Unlike the PLF phenotype in the beta frequency band, the non-PL power phenotype was not rescued in *Ntsr1*:cON mice as observed by the difference illustrated in the *Ntsr1*:cON – WT control plot (*Middle* panels; Fig. 4B and Supp. Fig. 4B) and by a lack of difference illustrated in the cON – *Ntsr1*:cON difference plot (*Right* panels; Fig. 4B and Supp. Fig. 4B). Therefore, deletion in *Ntsr1*-marked neurons is not required for this increase in single trial non-PL power in the low gamma band.

### 3.7. The increase in “resting” gamma band power observed in the Fmr1 KO is not reproduced in cON Fmr1 mice

**Fig. 5.**
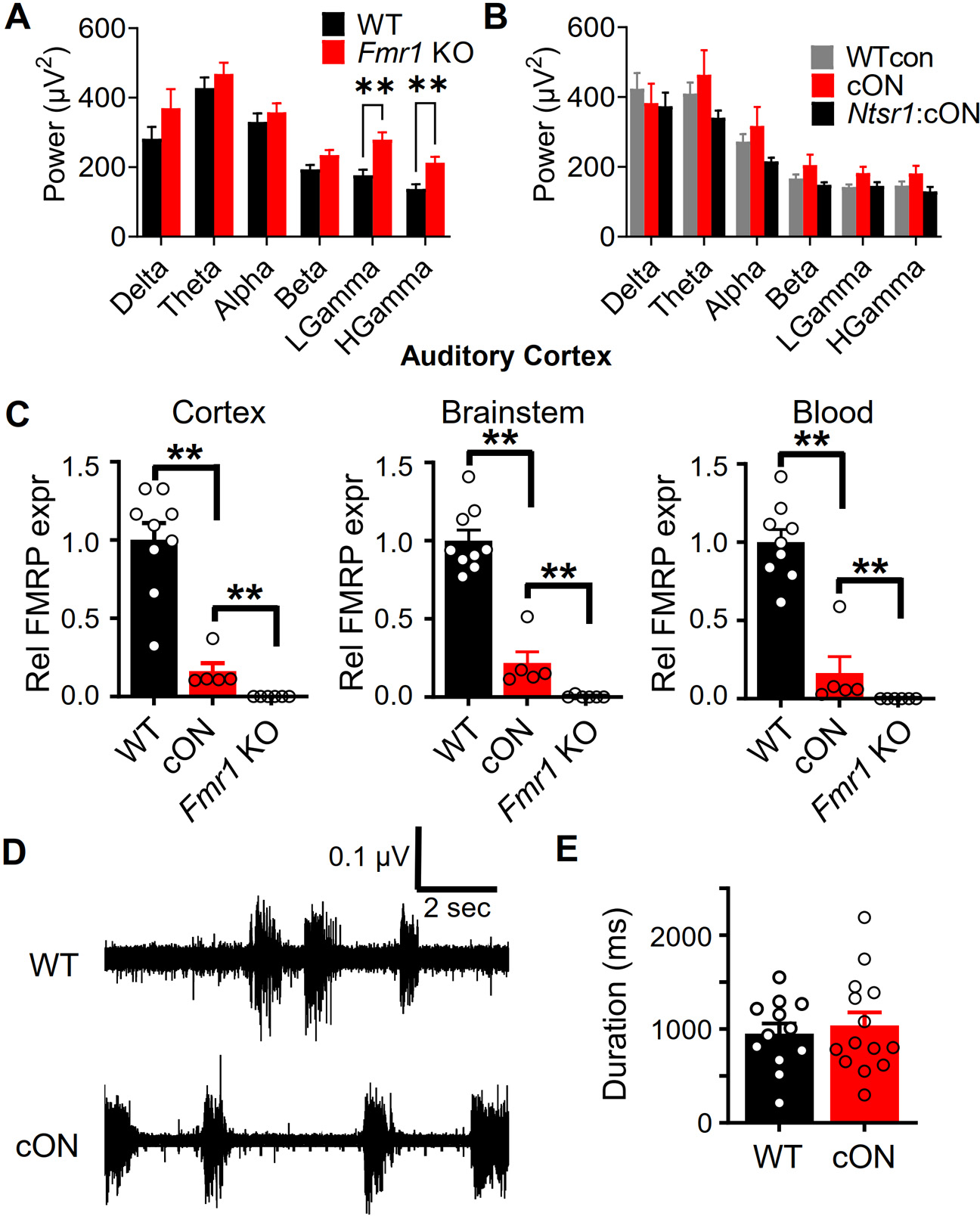
The inability to reproduce the increased resting power in the *Fmr1* KO in cON mice may be due to residual FMRP in the cortex. A) During rest, there was increased power in the gamma bands in *Fmr1* KO mice as measured in auditory cortex. B) No power phenotype was detected in cON *Fmr1* mice. C) Relative FMRP expression in cortex, brainstem, and blood. D) Sample traces from extracellular recording performed in acutely prepared cortical slices showing spontaneously occurring bursts of circuit activity. E) There was no difference in the duration of bursts between WT and cON mice. For A and B, n’s provided in Figs. 1 and 2. For C, *n* = 9,5,6 mice: WT,cON,*Fmr1* KO. For D and E, *n* = 12,17 slices from 3,4 mice; WT, cON. ** *p* < 0.01.

As previously reported, we observed higher gamma power in the *Fmr1* KO mouse during resting conditions – when no sound stimuli were being presented (Lovelace et al., 2018; Jonak et al., 2020; Wen et al., 2019; Sinclair et al., 2017) (Fig. 5A). We were unable to reproduce this phenotype in the cON mice under any conditions – 1) in auditory cortex (Fig. 5B), 2) in frontal cortex (Supp. Fig. 7), 3) or in young FVB mice (data not shown). Therefore, we were not able to use conditional expression to determine the role of *Fmr1* deletion in *Ntsr1*-marked neurons for the enhanced resting gamma power phenotype in *Fmr1* KO mice.

### 3.8. Remaining FMRP levels in cON mice may be sufficient to rescue, and thereby normalize, some phenotypes observed in the Fmr1 KO

While the PLF in frontal cortex and the resting EEG phenotypes observed in the *Fmr1* KO (Figs. 3 and 5, respectively) do not occur in cON mice, two other key *Fmr1* KO phenotypes in our study are reproduced in cON mice: 1) decreased PLF in the beta band during the chirp (see Fig. 2B, left), and 2) increase single trial non-PL power during the chirp (Fig. 4). Previous studies have shown that other phenotypes observed in the *Fmr1* KO remain in the cON using direct comparisons with a WT control (Gonzalez et al., 2019) or indirectly using other comparisons (Rais et al., 2021). Therefore, some phenotypes are reproduced and some are not.

The cON mice actually express about 5–15% the normal levels of FMRP in adult hippocampus (Dr. David Nelson, personal communication), but based on this small number and previous successful use of the cON mouse line, we assumed it was an effective KO control (Rais et al., 2021; Guo et al., 2011; Gonzalez et al., 2019). Taking this into account, we hypothesize that the lack of resting EEG and frontal cortex PLF phenotypes in cON mice is due to the sufficiency of remaining levels of FMRP to normalize the phenotypes.

To better support this hypothesis that residual *Fmr1* expression in the cON mouse is sometimes enough to block a phenotype observed in the *Fmr1* KO, we needed to measure FMRP protein levels in cortex and brainstem – two locations where *Fmr1* deletion has been demonstrated to induce EEG and audiogenic seizure phenotypes observed in the *Fmr1* KO (Lovelace et al., 2020a; Rais et al., 2021; Gonzalez et al., 2019; Hays et al., 2011). We also examined relative FMRP levels in blood to determine if they were similar to that in brain. Using an immunoassay for quantifying protein, we first found the anticipated undetectable relative levels of FMRP in the *Fmr1* KO. For the cON mice, relative FMRP levels were ~16% over all tissues and indistinguishable between cortex and brainstem (Fig. 5C). The reason for the one high value in the cON group across all tissues is unclear, but even if this point were removed, the statistical differences remain. In summary, our data indicate that remaining relative FMRP levels are essentially equal in cortex and brainstem, and that these levels may be enough to block some phenotypes and not others.

We wanted to determine if one robust slice electrophysiology phenotype that has been repeatedly observed is blocked by expression of remaining FMRP in cON mice. In slice preparations of somatosensory cortex, spontaneously occurring activity bursts interspersed between quiet periods occur (Sanchez-Vives and McCormick, 2000) (Fig. 5D). These bursts represent the synchronized activity of neurons at the recording site and are synchronized across the layers and horizontal distances of cortex on the scale of millimeters. The bursts in the *Fmr1* KO slices are longer in duration, and this phenotype is dependent upon *Fmr1* deletion in the cortex (Gibson et al., 2008; Hays et al., 2011). Indeed, no change in burst duration was observed in cON mice indicating that residual FMRP in the cortex was sufficient to block the phenotype (Fig. 5E).

## 4. Discussion

### 4.1. Main conclusion: Decreased phase-locked power in responses to sounds partly stems from Fmr1 deletion in brainstem circuits while non-phase-locked power does not

We tested the hypothesis that changes in sound responses in the *Fmr1* KO involves changes in brainstem circuitry. We did this by determining if *Fmr1* deletion in a subpopulation of brainstem neurons – *Ntsr1*-marked neurons – was necessary for decreased sound-driven phase-locking when employing a chirp stimulus. We demonstrated that deletion in this population was indeed necessary by the normalization, or rescue, of the phase-locking phenotype through conditional expression of *Fmr1* in an otherwise *Fmr1* KO mouse. This result was not generalized across all conditions. Rather, it was specific for: 1) recordings performed in the auditory cortex and 2) sounds modulated in the beta band (13–30 Hz).

We also hypothesized that the increase in single trial non-phase-locked power (non-PL power) in the gamma band that is observed in the *Fmr1* KO during the same chirp would not require *Ntsr1*-marked neurons. This was indeed the case. This is consistent with previous studies indicating that *Fmr1* deletion in cortex is sufficient to induce this phenotype (Lovelace et al., 2020a; Rais et al., 2021). Therefore, the decreased sound-driven phase-locking in the beta band and the increased non-PL power in the gamma band are likely due to changes in different brain circuits in the *Fmr1* KO.

### 4.2. Attribution of effects to Fmr1 expression in brainstem Ntsr1-marked neurons is most probable

*Ntsr1*-Cre mice express Cre not only in auditory nuclei of the brainstem, but also in a limited number of other brain regions (Table 1). This includes strong expression in pyriform and entorhinal cortices and in the olfactory bulb. Our assertion that *Fmr1* expression in *Ntsr1*-marked brainstem neurons was the most relevant to our experiments is based on three important points. First, these regions outside of the brainstem are not in the lemniscal auditory pathway which provides the most direct auditory information to the cortex. Second, a previous study observed that the decreased PLF in the beta band of a chirp is not due to *Fmr1* deletion in cortical forebrain neurons (Lovelace et al., 2020a; Rais et al., 2021) confirming our assumption that *Fmr1* expression in *Ntsr1*-marked cortical neurons – in pyriform and entorhinal cortices - were unlikely accounting for the PLF beta frequency decrease. And third, this logic was similarly applied in our previous study where we discovered that the audiogenic seizure phenotype in *Fmr1* KO mice requires deletion in *Ntsr1*-marked brainstem neurons (Gonzalez et al., 2019). And the localization to the brainstem in that study was consistent with past studies indicating that audiogenic seizures are mediated almost solely by brainstem circuits (Faingold, 2002; Ribak et al., 1994; Browning et al., 1999).

*Ntsr1*-marked neurons of the brainstem are most concentrated in the inferior colliculus (IC), but there is also expression in the cochlear root nucleus and in a subpopulation of ventral cochlear neurons. We speculate that deletion in the inferior colliculus most likely accounts for the decreased phase-locking in the beta band observed in the *Fmr1* KO mouse. Future studies with other Cre mouse lines would be needed to establish this link more strongly.

### 4.3. The mechanisms of dysfunction in brainstem neurons causing the phase-locking deficit remain unknown

We have provided candidate circuits that could underlie altered sensory responses in the *Fmr1* KO mouse – principally the IC and/or a subpopulation of cochlear nucleus neurons. We have not addressed the mechanisms. Neurons in the inferior colliculus have wider frequency tuning curves and fire more strongly in response to sound in the *Fmr1* KO mouse (Nguyen et al., 2020). It is most likely that *Ntsr1*-marked neurons in the inferior colliculus have these changes since they represent most of the glutamatergic neurons in the IC (Gonzalez et al., 2019). These response changes in IC neurons could underlie the deficits in cortical sound-driven phase-locking that is observed in *Fmr1* KO mice and FXS individuals. Future work will be needed to determine the characteristics of local circuits in the inferior colliculus of *Fmr1* KO mice to determine the mechanisms underlying the PLF decrement phenotype.

Other auditory regions immediately presynaptic or postsynaptic to *Ntsr1*-marked neurons may also be involved since *Fmr1* expression in either presynaptic or postsynaptic neurons regulates synaptic function and connectivity (Pfeiffer and Huber, 2007; Patel et al., 2014; Patel et al., 2013; Zhang et al., 2021; Deng et al., 2013). For example, nuclei of the lateral lemniscus and the lateral superior olive project to and receive a projection from the IC (Pickles, 2015). Therefore, changes in function of these recurrent circuits induced by *Fmr1* re-expression in the IC could underlie the rescue of the phase-locking phenotype even though these other structures lack re-expression. Finally, long-term changes in activity levels and patterns in the IC may induce adaptations in brain regions that are multiple synapses removed, such as the auditory cortex.

### 4.4. Effectiveness of the NECESSITY strategy using the conditional ON (cON) mice

We chose to target cell subpopulations by testing for *necessity* because we thought it was easier and more controlled. In a study examining the audiogenic seizure phenotype in *Fmr1* KO mice, this approach produced a positive result while testing for *sufficiency* using conditional deletion produced a negative result (Gonzalez et al., 2019). Therefore, we assumed it would be easier to obtain a positive, useful result testing for *necessity* in the context of the PLF phenotype. This approach is also better controlled by providing both WT and *Fmr1* KO littermate controls for comparison to *Fmr1* expression effects. This is not possible for the *sufficiency* approach using conditional deletion where only WT controls exist (Gonzalez et al., 2019).

Our data indicate that Cre induced *Fmr1* expression occurred in cells that naturally express *Fmr1* and that the levels of *Fmr1* in individual cells were probably close to that in WT. But *Fmr1* induced using *Ntsr1*-Cre mice may occur at a slightly older embryonic stage compared to WT. The earliest observation of expression of FMRP is around E14.5 (Casingal et al., 2020), and we found that Cre-expression in the *Ntsr1*-Cre line begins sometime before birth (Supp. Fig. 1). Based on this, our re-expression in conditional expression experiments may occur up to 10 days after WT expression begins. Therefore, while we interpret a failure of conditional *Fmr1* expression in *Ntsr1*-marked neurons to rescue the STP phenotype (see Fig. 4) as indicating no role for these neurons, this may not be the case. *Ntsr1*-marked neurons may actually be able to rescue, but we don’t conditionally express *Fmr1* early enough.

It is possible that low FMRP levels early in development induce *secondary* abnormal developmental mechanisms to compensate for changes that are more *primary* to *Fmr1*-expression loss. The compensation would help normalize or mask these primary mechanisms. Therefore, important roles for *Fmr1* that impact EEG alterations may be masked when observing the *Fmr1* KO or the cON mouse. Because of this, *Fmr1* re-expression may not normalize some phenotypes and may even induce unexpected changes not observed in either cON or WT mice. This is observed in Fig. 2A where *Ntsr1*:cON mice have unusually strong phase-locking in the low-gamma frequency band during the chirp. In this panel, neither the WT nor cON mice have this property. But our two main findings regarding phase-locking and non-phase-locked power did not have this property, and thus, these data were interpretable in terms of a rescue.

In summary, the *necessity* approach did produce positive results. But overall, the phenotypes were weaker in the cON mice compared to *Fmr1* KO mice limiting our ability to more comprehensively determine the role of *Ntsr1*-marked neurons in *Fmr1* KO EEG phenotypes.

### 4.5. Different phenotypes in Fmr1 KO mice require different levels of FMRP loss

While minimum rates of FMRP mosaicism (20%) and levels (50%) have been reported to rescue particular phenotypes in cell culture, no data exist at the systems level (Graef et al., 2020; Nakamoto et al., 2007). Here, we report that a minimum FMRP expression level of ~16% of normal is sufficient to rescue many of the EEG phenotypes that we clearly observed in the *Fmr1* KO. We also make the same observation for a circuit excitability phenotype we observe in an *ex vivo*, acute brain slice preparation.

If an *Fmr1* KO phenotype was not observed in the cON *Fmr1* mouse, this would suggest that the minimum FMRP level to rescue the phenotype was below 16% of normal. If a phenotype was reproduced in the cON mouse, this would suggest that the minimum FMRP level for rescue was greater than 16%. One caveat with this conclusion is that we cannot be certain that the cON mouse strain would even display the rescued phenotypes if we performed a control experiment by removing the remaining FMRP in those mice. We used the *Fmr1* KO line as this control, but there could be a genetic background difference that could confound effects of FMRP levels. To reduce this possibility, we backcrossed both lines to C57Bl/6 J mice from Jackson labs for at least 8 generations to promote genetic background similarity among all C57bl/6 J mice used in this study (see Methods, *Mice*).

Because we did not measure FMRP levels in the cON *Fmr1* mice at different points during development, we cannot state whether the rescue of the phenotype is due to residual FMRP expression maintained during development or due to more acute gain of FMRP expression in the adult. Therefore, we don’t know the relevant timepoints at which residual FMRP levels rescue phenotypes. We did not measure FMRP levels in FVB cON mice so the reasons for not observing robust EEG phenotypes in these mice are likely similar, but this was not established.

How might our results in mice be linked to FXS individuals? It is important to note that decreased FMRP levels in FXS individuals are largely due to mosaicism (Hagerman et al., 2017). There are no known mutations that induce a more uniform decrease in FMRP across all cells. But in the cON *Fmr1* mouse, there is most likely a uniform decrease in FMRP levels among all cells. One scenario when levels of FMRP may be increased at relatively uniform levels among neurons is viral delivery of the *Fmr1* gene in humans. Our data suggest that reinstating low-levels of FMRP may be sufficient for alleviating certain symptoms – perhaps useful knowledge if technical issues limit the amount of re-instatement of FMRP in certain cell types or if it is desired to avoid over-expression in existing FMRP-positive cells in mosaic FXS individuals.

### 4.6. Relevance to ASD in general

Many neurodevelopmental disorders (NDD), including FXS and the wider class of autism, display hypersensitivity to sounds (Musumeci et al., 1994; Miller et al., 1999; Baranek et al., 2008; Ben-Sasson et al., 2009; Hagerman et al., 2009). For the *Fmr1* KO, this comes in the form of sound induced seizures which is likely mediated by IC dysfunction (Gonzalez et al., 2019). Some ASD individuals with *SYNGAP1* and *UBE3A* mutations also have some form of auditory hypersensitivity (Walz and Baranek, 2006; DRM et al., 2019), and the corresponding mouse models also have audiogenic seizures (Jiang et al., 1998; Clement et al., 2012). Interestingly, decreased phase-locking to auditory stimuli is also observed in *SYNGAP1* patients (Cote et al., 2021). Since both auditory hypersensitivity and decreased phase-locking exists with both *FMR1* and *SYNGAP1* loss-of-function, this suggests that the hypersensitivity and decreased phase-locking might be linked in autism and that *SYNGAP1* may have a similar function in the inferior colliculus as *Fmr1*.

### 4.7. Brainstem sensory circuit changes impact potential biomarkers for Fragile X syndrome

The decreases in PLF and increase in non-PL power observed in the *Fmr1* KO mouse are also observed in EEG recordings of humans with FXS (Ethridge et al., 2017). Our results are the first to indicate that brainstem circuit changes may play a role in these potential biomarkers. By virtue of rescuing a phase-locking decrease by targeting a very limited neuronal population, we also demonstrate that targeting that population could be an effective strategy for alleviating sensory symptoms in FXS. By finding candidate sites for circuit dysfunction that can underlie altered sensory processing, we now have the ability to study the detailed mechanisms for this specific deficit in FXS and potentially develop novel therapeutic strategies.

## Supplementary Material

1

## Figures and Tables

**Table 1:** Ntsrl-cre expression.

Brain Structures	Expression Strength

Inferior Colliculus	3+
VCN/CRN	2+
Ventral Pons	0–1
Pyr, Ent, Sub	2–3+
Olfactory Bulb	3
Superficial Layer of Superior Colliculus	2
Deep Layer of Superior Colliculus	1
Cerebellum	1−
Periaqueductal Gray	1
Amygdala	0–1
Thalamus	0
Neocortex	0

3 = Strong, 2 = Moderate, 1 = Sparse, 0 = None. Predicted neuronal cell type: − = inhibitory, + = excitatory. VCN = ventral cochlear nucleus, CRN = cochlear root nucleus, Pyr = pyriform cortex, Ent = entorhinal cortex, Sub = subiculum. Structures directly involved in audition in gray. Based on references (Gong et al., 2007; GENSAT, 2003).

## Abbreviations:

FXS: Fragile X Syndrome
EEG: electroencephalography
KO: knockout
FMRP: Fragile X mental retardation protein
IC: inferior colliculus
PLF: phase-locking factor
