# Supplementary material for: A sound-driven cortical phase-locking change in the Fmr1 KO mouse requires Fmr1 deletion in a subpopulation of brainstem neurons
Source: Neurobiol Dis. Author manuscript; Available in PMC 2022 Aug 1. (PMC9273231; doi:10.1016/j.nbd.2022.105767)
Supplement: 1 [file NIHMS1818743-supplement-1.pdf]

A

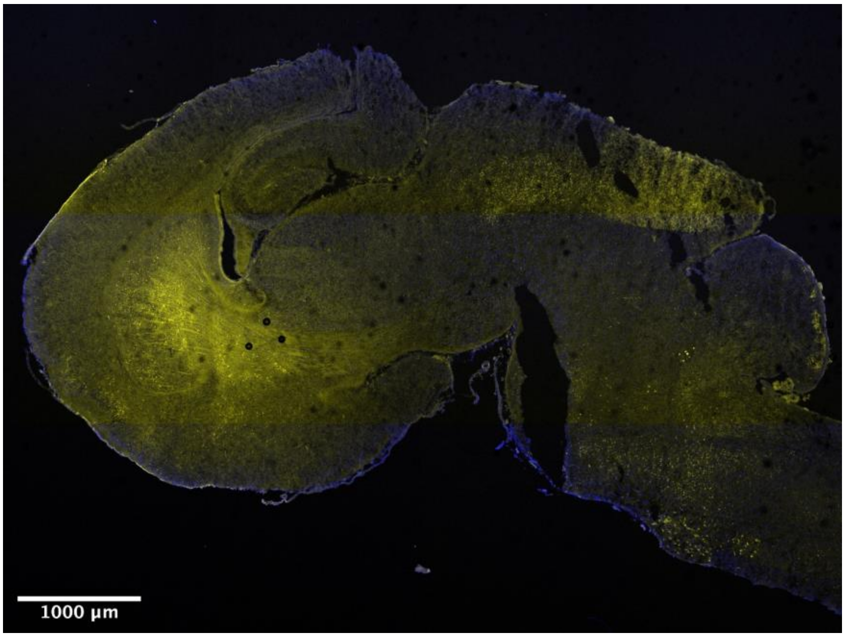

B

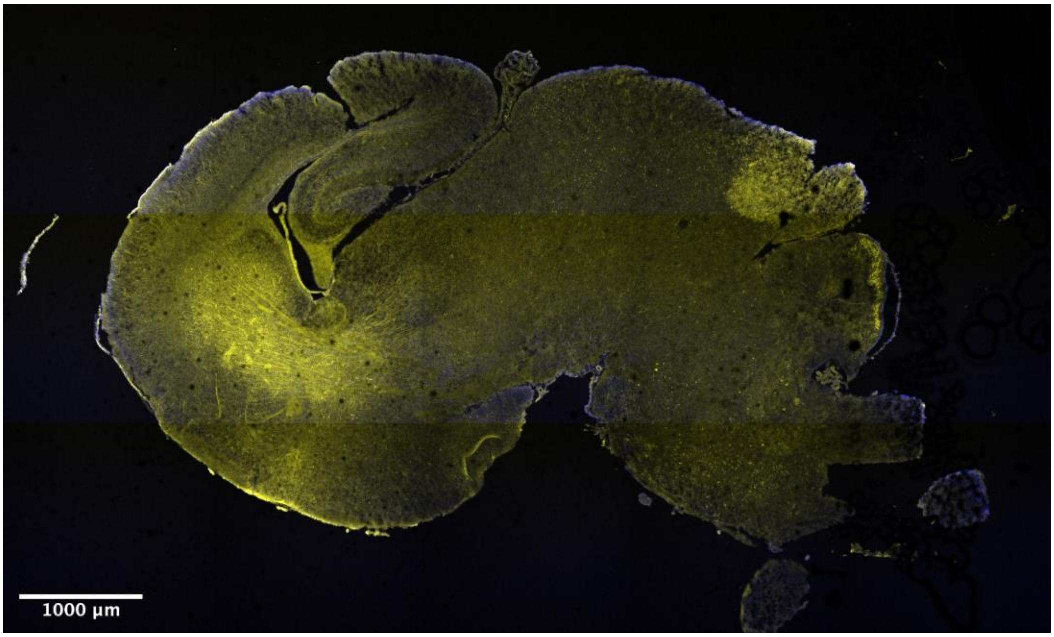

Supplemental Figure 1. YFP reporter images for *Ntsr1*-Cre expression at P0. Mice were perfused and fixed in formalin then post-fixed in 30% sucrose before sectioning into 50 µm slices on a cryotome. Sagittal sections are shown. The bottom section is more lateral.

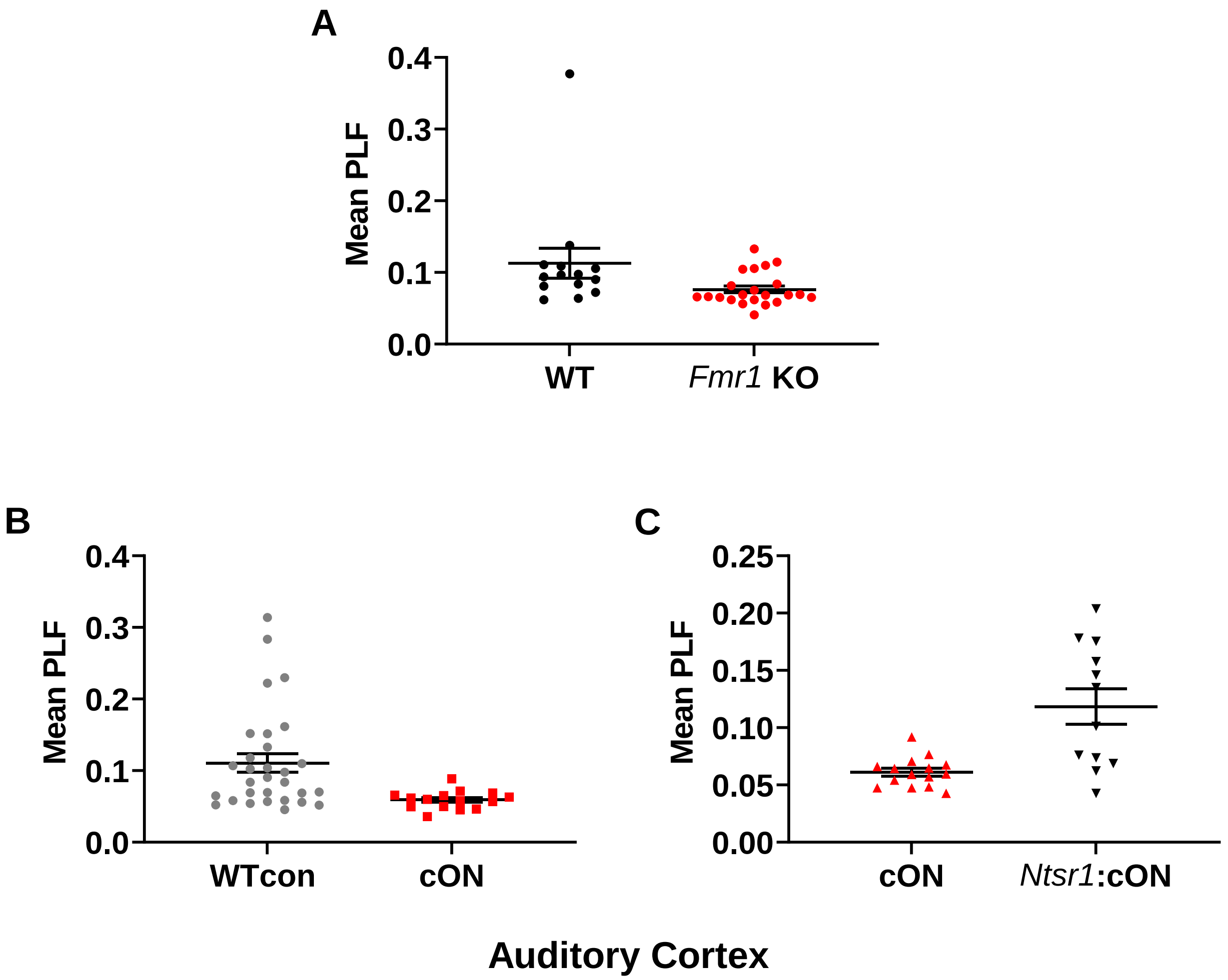

Supplementary Figure 2. Scatterplots of PLF obtained from the Beta frequency band depicted in color plots of Figures 1 and 2. The average PLF was obtained from the statistically significant clusters marked in the difference plots and found within the beta frequency band. **A**) From Figure 1, WT and *Fmr1* KO mice (n=17,20; WT,KO), **B**) From 1st plot Fig. 2B, Wtcon and cON mice, and **C**) rom 3rd plot Fig. 2B, cON and Ntsr1:cON mice. (n=29,25,12; WTcon,cON,*Ntsr1*:cON).

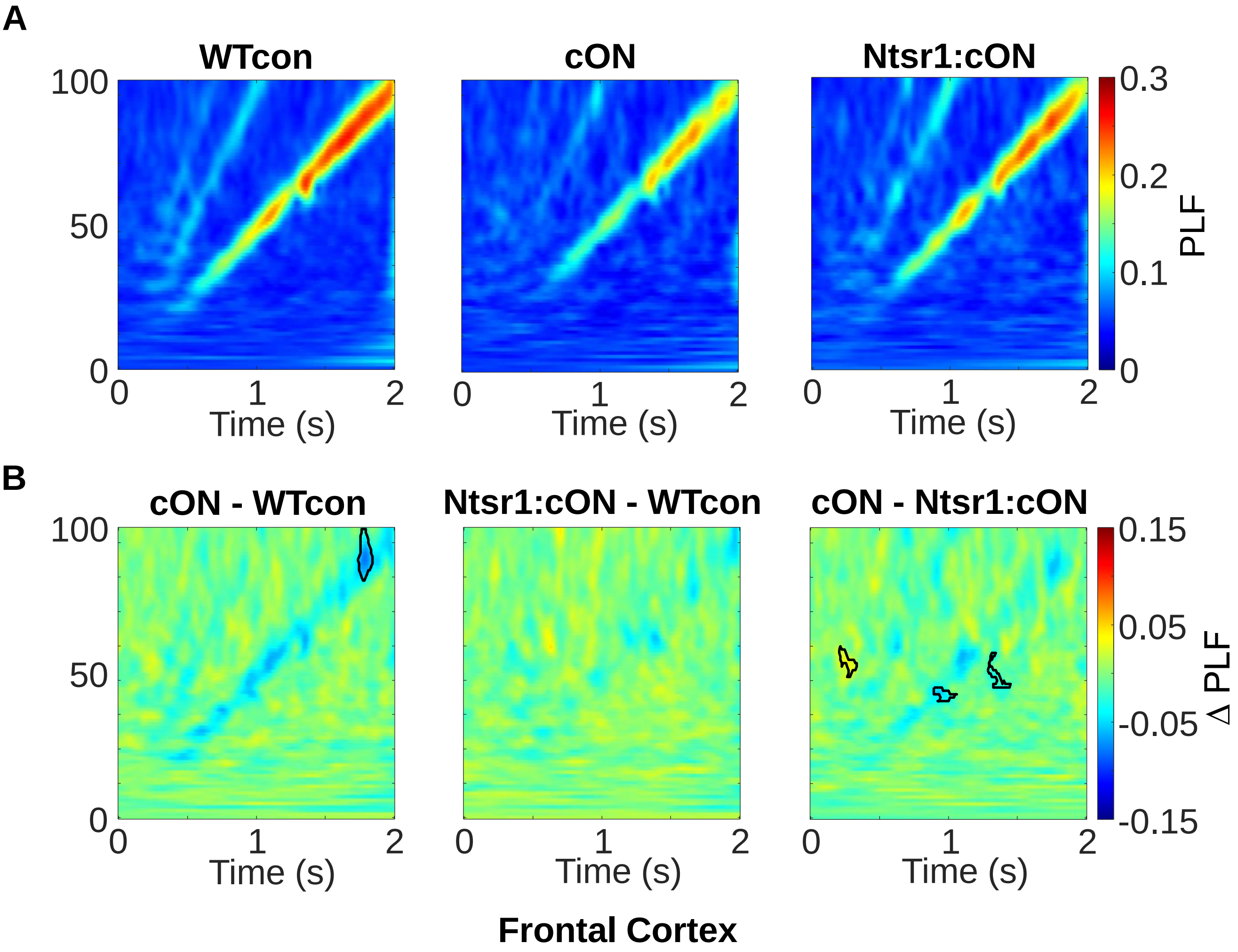

Supplementary Figure 3. There is a decreased PLF phenotype in the high gamma frequency band in frontal cortex of 4 week old cON mice of the FVB strain. A) Average PLF color plots. B) The difference plot based on plots in A. In the far left plot, bold black lines indicate a decreased PLF in the upper high gamma frequency band measured in cON mice. n=17,10,11 mice; WTcon,cON,*Ntsr1*:cON. * p<0.05, ** p<0.01,

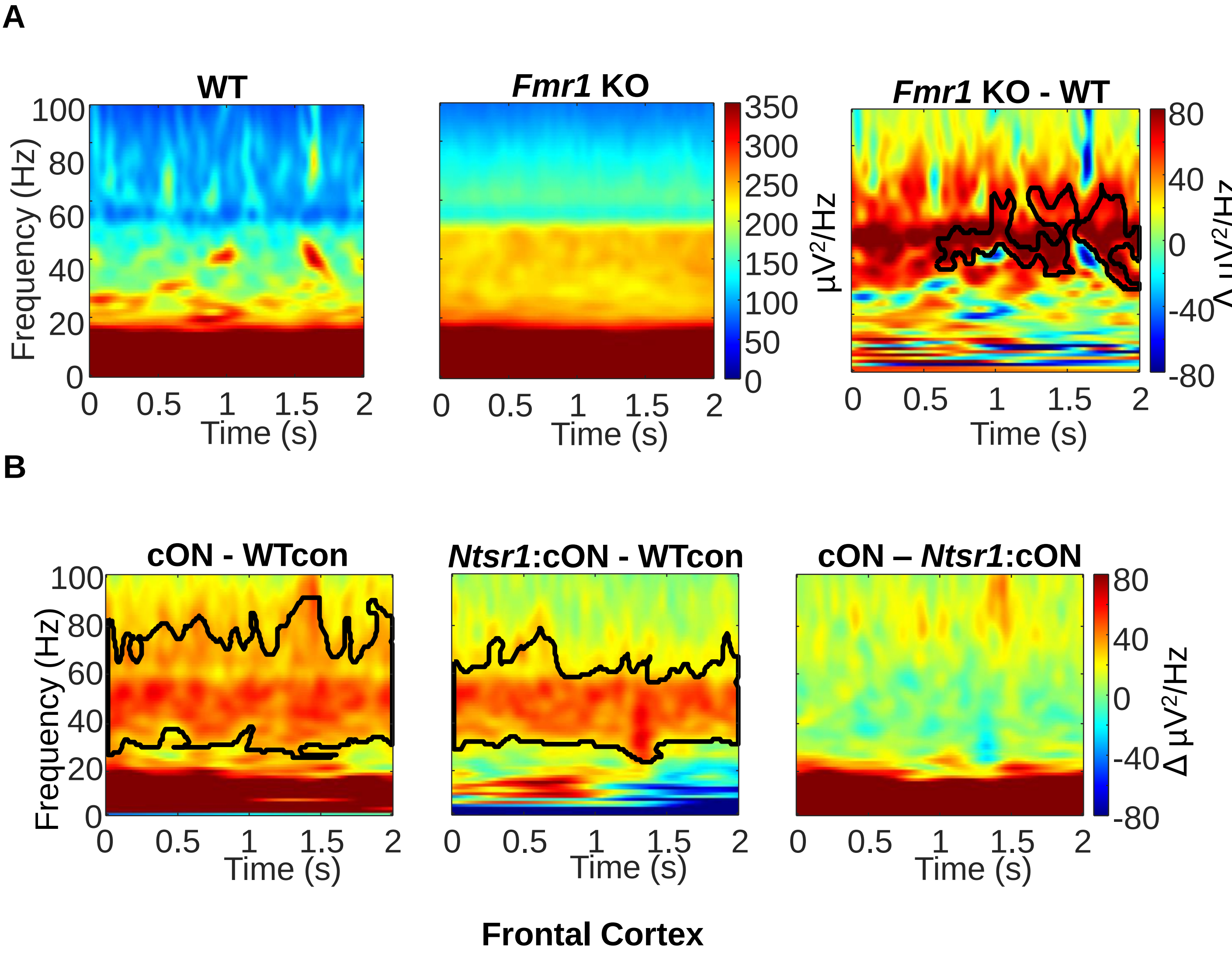

Supplementary Figure 4. An increase in non-phase-locked power observed in frontal cortex of the *Fmr1* KO was not rescued by *Fmr1* re-instatement in *Ntsr1*-marked neurons. A) Average single trial non-phase-locked (non-PL) power plots (*left*, *middle*) and difference plot (*right*) for WT and *Fmr1* KO mice. A decreased non-PL power is observed at gamma frequencies in the *Fmr1* KO (n=17,20 mice; WT,KO). B) Average difference non-PL difference plots obtained from auditory cortex in conditional expression experiments. The cON *Fmr1* mice had a phenotype of increased power in the low gamma frequency band (*left*) which was not rescued by re-instatement of *Fmr1* in *Ntsr1*-marked neurons (*middle, right*) (n= 29,15,12 mice; WTcon,cON,*Ntsr1*:cON). Chirp in all plots used the 14 kHz sound carrier. Dark lines in difference plots border regions of statistically significant difference.

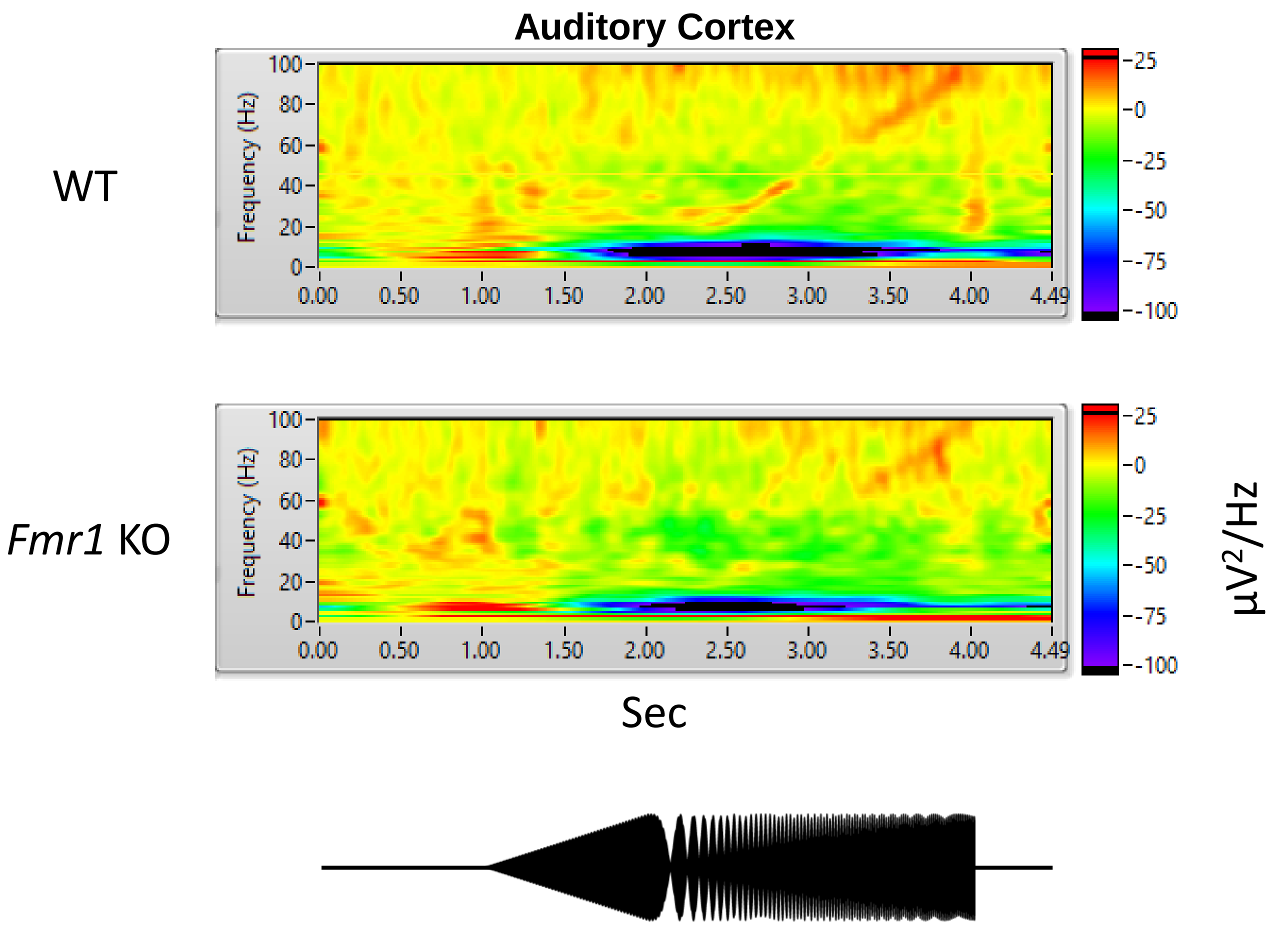

Supplementary Figure 5. Average background subtracted non-PL power of the complete ramp/chirp trial is not statistically different between *Fmr1* KO and WT mice in auditory cortex. Background was measured in the first second of the trial during which no sound was presented. Subtraction was done independently for each frequency. Below, the sound stimulus is aligned with the non-PL power color plots. For WT mice, the power in the high gamma band was significantly increased during the sound stimuli when compared to the background period, but no difference between WT and *Fmr1* KO was detected over the same epoch (data not shown). While the diagonal corresponding to the increasing modulation during the chirp appears lower in the *Fmr1* KO, this was not statistically significant. n=17,20 mice; WT,KO

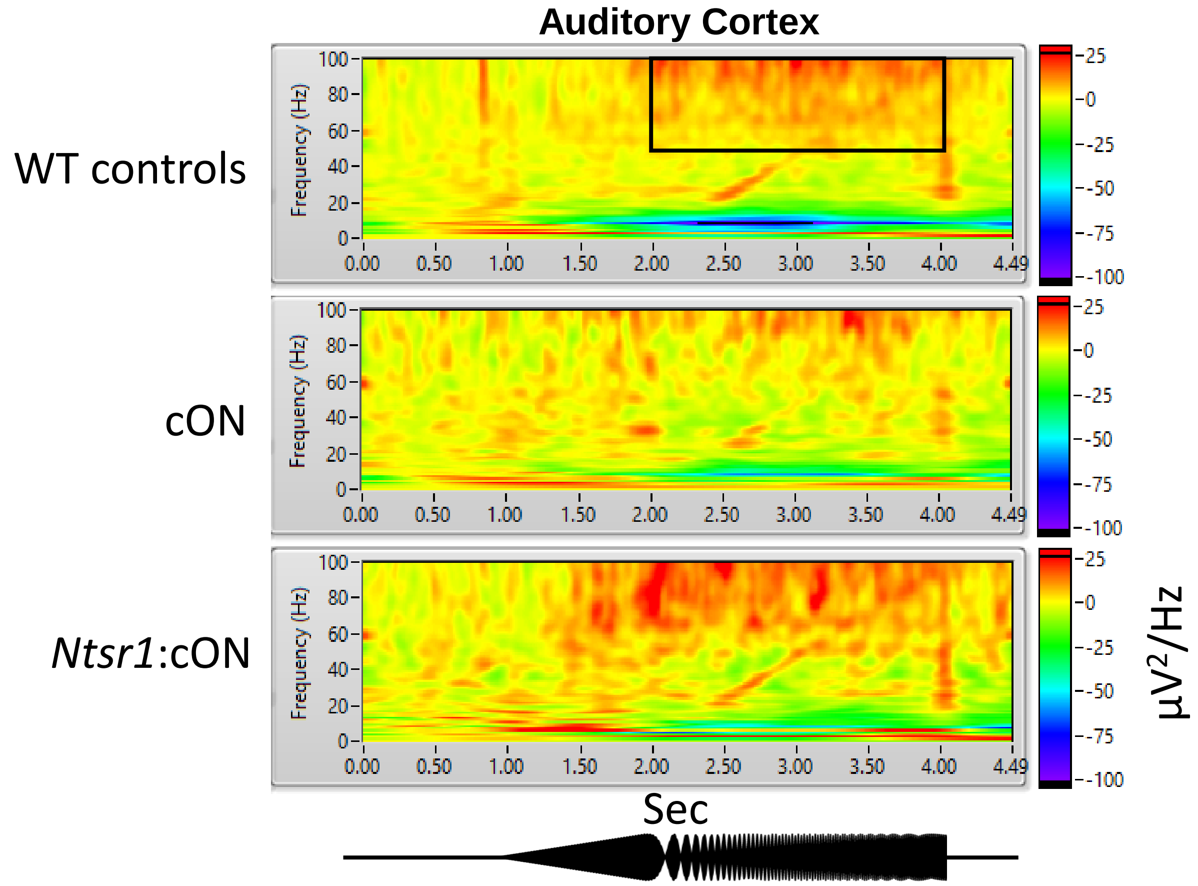

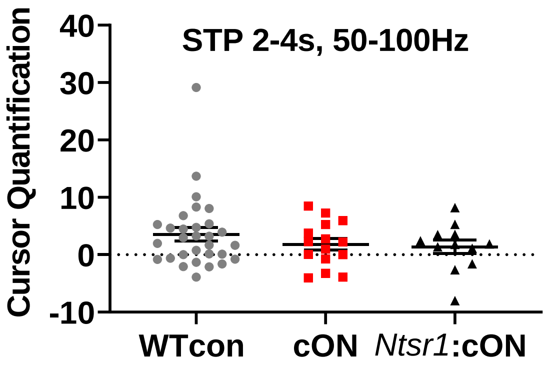

Supplementary Figure 6. Average background subtracted non-PL power of the complete ramp/chirp trial is not detectably different between any genotypic groups in conditional *Fmr1* expression experiments in auditory cortex. Background was measured in the first second of the trial during which no sound was presented. Subtraction was done independently for each frequency. Below, the sound stimulus is aligned with the non-PL power color plots. For all genotypic groups, the power in the high gamma band was significantly increased during the chirp when compared to the background period (data not shown) (n= 29,15,12 mice; WTcon,cON,*Ntsr1*:cON). Bottom: The variability of responses was assessed within each group to produce a single average STP value for each mouse from 2-4 s between 50-100 Hz (black box on top) and further indicates no difference between groups in non-PL power.

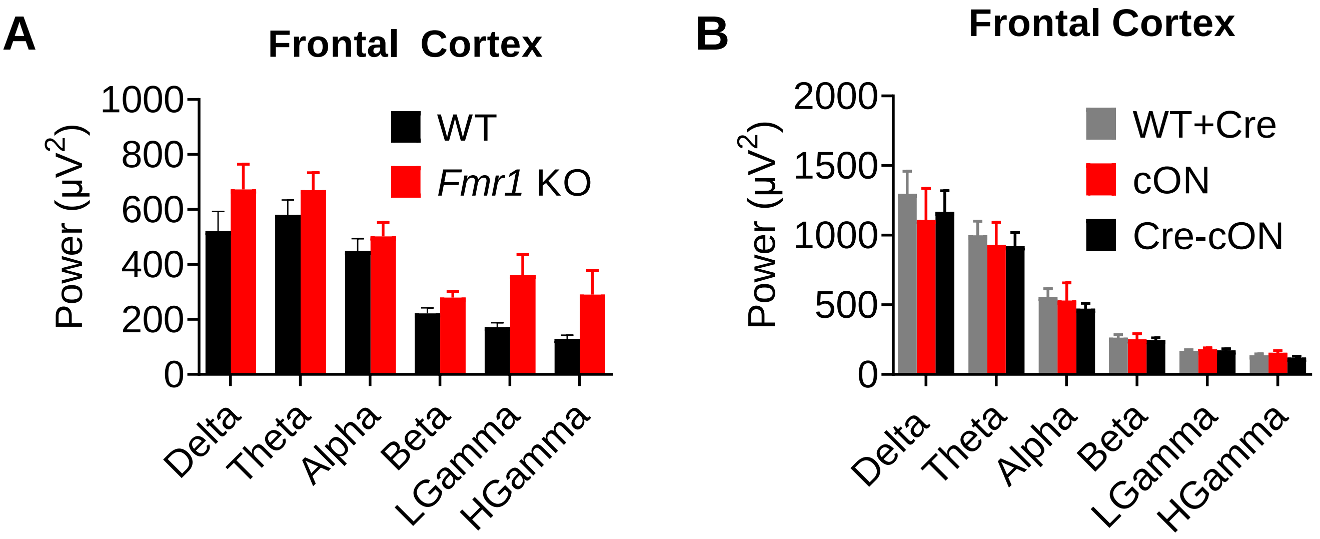

Supplementary Figure 7. A statistical trend of increased low gamma power (p=0.13) of the resting EEG in the *Fmr1* KO observed in frontal cortex. No detectable changes in cON mice compared to WT controls. A) Average resting EEG power in the standard frequency bands for *Fmr1* KO and WT mice (N=17,20 mice; WT,KO). B) Average resting EEG power in the standard frequency bands for the conditional expression experiment (n=29,15,12 mice; Wtcon,cON,*Ntsr1*:cON).
